# Engagement With a Relaxation and Mindfulness Mobile App Among People With Cancer: Exploratory Analysis of Use Data and Self-Reports From a Randomized Controlled Trial

**DOI:** 10.2196/52386

**Published:** 2024-05-31

**Authors:** Sonja Schläpfer, Fabian Schneider, Prabhakaran Santhanam, Manuela Eicher, Tobias Kowatsch, Claudia M Witt, Jürgen Barth

**Affiliations:** 1 Institute for Complementary and Integrative Medicine University Hospital Zurich and University of Zurich Zurich Switzerland; 2 Centre for Digital Health Interventions Department of Management, Technology, and Economics ETH Zurich Zurich Switzerland; 3 Institute of Higher Education and Research in Healthcare Faculty of Biology and Medicine University of Lausanne and Lausanne University Hospital Lausanne Switzerland; 4 Department of Oncology Lausanne University Hospital Lausanne Switzerland; 5 Institute for Implementation Science in Health Care University of Zurich Zurich Switzerland; 6 School of Medicine University of St.Gallen St.Gallen Switzerland

**Keywords:** mobile health, mHealth, digital health, eHealth, smartphone, mobile phone, implementation, adherence, self-guided, unguided, fully automated, conversational agent, chatbot, behavior change, tailoring, self-care, cancer, app development

## Abstract

**Background:**

Mobile health (mHealth) apps offer unique opportunities to support self-care and behavior change, but poor user engagement limits their effectiveness. This is particularly true for fully automated mHealth apps without any human support. Human support in mHealth apps is associated with better engagement but at the cost of reduced scalability.

**Objective:**

This work aimed to (1) describe the theory-informed development of a fully automated relaxation and mindfulness app to reduce distress in people with cancer (CanRelax app 2.0), (2) describe engagement with the app on multiple levels within a fully automated randomized controlled trial over 10 weeks, and (3) examine whether engagement was related to user characteristics.

**Methods:**

The CanRelax app 2.0 was developed in iterative processes involving input from people with cancer and relevant experts. The app includes evidence-based relaxation exercises, personalized weekly coaching sessions with a rule-based conversational agent, 39 self-enactable behavior change techniques, a self-monitoring dashboard with gamification elements, highly tailored reminder notifications, an educational video clip, and personalized in-app letters. For the larger study, German-speaking adults diagnosed with cancer within the last 5 years were recruited via the web in Switzerland, Austria, and Germany. Engagement was analyzed in a sample of 100 study participants with multiple measures on a micro level (completed coaching sessions, relaxation exercises practiced with the app, and feedback on the app) and a macro level (relaxation exercises practiced without the app and self-efficacy toward self-set weekly relaxation goals).

**Results:**

In week 10, a total of 62% (62/100) of the participants were actively using the CanRelax app 2.0. No associations were identified between engagement and level of distress at baseline, sex assigned at birth, educational attainment, or age. At the micro level, 71.88% (3520/4897) of all relaxation exercises and 714 coaching sessions were completed in the app, and all participants who provided feedback (52/100, 52%) expressed positive app experiences. At the macro level, 28.12% (1377/4897) of relaxation exercises were completed without the app, and participants’ self-efficacy remained stable at a high level. At the same time, participants raised their weekly relaxation goals, which indicates a potential relative increase in self-efficacy.

**Conclusions:**

The CanRelax app 2.0 achieved promising engagement even though it provided no human support. Fully automated social components might have compensated for the lack of human involvement and should be investigated further. More than one-quarter (1377/4897, 28.12%) of all relaxation exercises were practiced without the app, highlighting the importance of assessing engagement on multiple levels.

## Introduction

### Background

Mobile health (mHealth) apps offer unique opportunities to deliver self-care interventions and support behavior change, but poor user engagement and retention rates pose substantial challenges. mHealth apps are a convenient approach to facilitate behavior change with the potential to reach large numbers of people [1-3]. However, in the same manner that mHealth apps provide easy access with a low barrier to start an intervention, they also provide a low barrier to stop using an intervention, turning a great advantage of mHealth apps into a fundamental challenge [2]. Low engagement is problematic because mHealth apps that support healthy behaviors can only be effective if people take an active role, learn the necessary skills to change their behavior, and apply the skills to everyday life, making engagement a pivotal prerequisite to health behavior change [4-8]. In studies using mHealth apps, poor engagement can also confound the outcome and impact the validity of the results as study dropouts may differ from completers [2,9]. While many mHealth apps have significant issues with sustained engagement [10-17], this is particularly true for fully automated mHealth apps without any human support, also termed unguided or self-guided mHealth apps. A high level of human support in guided mHealth apps is typically associated with better engagement rates but at the cost of reduced scalability [18,19]. Hence, to increase the effectiveness of behavior change interventions and improve mHealth studies, it is critical to better understand what makes people stay engaged with mHealth apps [2,20-22] and especially with fully automated mHealth apps as the latter are more likely to be disseminated widely [23].

User engagement has been conceptualized differently across disciplines, but there is a consensus that engagement with an mHealth app needs to be examined on different levels [7,24]. The different levels stem from the crucial distinction between moment-to-moment engagement with the intervention at the *micro* level (ie, app use and user experience) and engagement with the broader intervention goal at the *macro* level (ie, target behavior) [5,7]. The micro and macro levels are closely interlinked, and engagement at the different levels can vary over time [5]. For example, during the initial use phase of mHealth apps, moment-to-moment engagement with the app may serve as preparation for behavior change. In a later phase, when people apply the skills they learned to everyday life, use of the app may no longer be required for engagement with the targeted behavior. Hence, reduced app use could be a sign of success rather than failure [2], highlighting the importance of comprehensively assessing engagement.

Most mHealth studies assess engagement with system use data at the micro level but do not consider engagement measures at the macro level. At the micro level, system use data such as the number of log-ins or the amount and type of content used are frequently applied as the only measure of engagement with an mHealth app. However, although system use data undoubtedly provide valuable information on certain aspects of microlevel engagement, these data are not considered a valid measure of engagement on their own [24]. Greater efforts are needed to combine different data sources, such as pairing system use data with self-report data or qualitative methods, to better understand the user experience [5,6,17,24]. At the macro level, assessing engagement remains a challenge and is often neglected in mHealth studies. To support research in this area, recent reviews have provided a valuable overview of available measures for exploring engagement in the behavior change process in daily life [5,6,24]. The listed measures to assess macrolevel engagement include sensor data to track behavior in real-life settings, analysis of social media patterns, and the repeated assessment of psychological constructs that are hypothesized to be important determinants of behavior change (eg, self-efficacy) [24]. Changes over time in psychological constructs such as self-efficacy could indicate engagement in the behavior change process [24]. Given the complexity of engagement as a construct, other measures of macrolevel engagement might be useful depending on the specific research context. Thus far, little research has been conducted applying these or other measures at the macro level of engagement and exploring their use in an mHealth behavior change setting [24].

### Objectives

We examined engagement at both a micro and a macro level with a newly developed relaxation and mindfulness app to reduce distress in people with cancer (CanRelax app 2.0) within a fully automated randomized controlled trial (RCT) over 10 weeks. The CanRelax app 2.0 is based on a first app version piloted in a feasibility study [25] and now includes more relaxation resources, a conversational agent, gamification elements, and 39 behavior change techniques (BCTs) translated into designed app features. The aims of this paper were to (1) describe the theory-informed development of the CanRelax app 2.0, (2) describe engagement with the app over 10 weeks as total app use and user feedback (micro level) and as self-efficacy and reported relaxation practices without using the app (macro level), and (3) examine whether engagement was related to user characteristics.

## Methods

### Study Design

The presented data originated from a larger RCT with an additional nonrandomized third arm. The study aimed to evaluate the effectiveness of the CanRelax app 2.0 in reducing distress in people with cancer who experience high distress compared with a waitlist control group. The primary end point was distress after 10 weeks assessed using the Patient Health Questionnaire Anxiety and Depression Scale [25]. Secondary outcomes were well-being (5-item World Health Organization Well-Being Index [26]), self-regulation (Multidimensional Assessment of Interoceptive Awareness Self-Regulation subscale [27]), and the course of distress over time (4-item Patient Health Questionnaire [28] and Distress Thermometer [29]; Multimedia Appendix 1 [25-31]). Eligible participants who self-reported high distress at baseline (Distress Thermometer score of ≥5 [29]) were randomized using 1:1 block randomization stratified by sex; those who self-reported low distress at baseline (Distress Thermometer score of <5 [29]) were included in a third arm as a nonrandomized intervention group to further explore user engagement. This nonrandomized intervention group received immediate access to the app (the same app as the randomized intervention group); the waitlist control group received full access to the app after 10 weeks. All groups were allowed to continue usual care and other interventions (including self-care interventions) as needed. As per sample size calculation, the target sample size was 210 participants in the randomized study arms (105 per arm); the sample size was not predefined for the nonrandomized third arm. The study was registered a priori at the German Clinical Trials Register (DRKS00027546; registration date: February 23, 2022). For this paper, data were taken from participants randomly assigned to the intervention group and participants assigned to the nonrandomized third arm. Further information on the study design and assessments is provided in Multimedia Appendix 1 [25-31]. The results of the RCT will be reported elsewhere.

### Inclusion Criteria

People were eligible to participate in the study if they (1) had received a cancer diagnosis within the last 5 years regardless of the type of cancer or stage at diagnosis, (2) were aged ≥18 years, (3) were fluent in German, (4) had a smartphone with regular internet access, and (5) gave informed consent to participate in the study. The exclusion criteria were suicidal ideation and known pregnancy according to participants’ self-reports. For this study, we analyzed an exploratory sample of the first 100 study participants who received full access to the CanRelax app 2.0 at inclusion. This corresponds to the sample needed to detect a meaningful difference (effect size *d*=0.8) in engagement between subgroups (high, 67/100, 67%, vs low, 33/100, 33%, distress) with a power of 0.95 (α=.05). The study was advertised for distressed individuals with cancer. Hence, we expected more high-distress than low-distress participants and assumed a ratio of approximately 2:1. Participants were excluded from the analysis if they withdrew from the study and requested that we exclude their data. In these cases, we included the next participant who received full access to the app at inclusion so that we had data from 100 participants for analysis.

### Recruitment Procedure

We launched the app in July 2022 through the Apple App Store and Google Play Store in Switzerland, Germany, and Austria. At the same time, we established a project website to facilitate recruitment. The website presented a summary of the study with key information such as the eligibility criteria, pictures of the app, and audio samples. It also included QR codes containing web links to the CanRelax app 2.0 in both app stores. We used social media sites (ie, Facebook, Twitter, and LinkedIn) and more traditional approaches (eg, consultations with health care providers, printed flyers, newsletters, and a press release by the University Hospital Zurich) to recruit study participants. Interested individuals could download the app free of charge and start by completing the app onboarding process as a first introduction to the app and the study. From the beginning, users were explicitly informed that they were interacting with a conversational agent, not a person. All study processes were fully automated; screening questions, study information and consent, enrollment, data collection, and all steps up to completion of follow-up were managed entirely through the CanRelax app 2.0. Participants had no contact with the research team at any time during the study unless they contacted the research team to ask questions before consenting or in case of technical issues. The RCT completed recruitment successfully in February 2023. Data collection was ongoing at the time of writing this paper.

### Intervention

#### Overview

The intervention was a fully automated mHealth app designed specifically to improve distress in adults with cancer through one type of self-care behavior (relaxation). Participants had access to the CanRelax app 2.0 over 20 weeks (10 weeks of intervention and 10 weeks of follow-up). On day 1, participants selected an outcome goal from a 5-item list in the app, including “find inner peace” (default if no choice was made), “improve coping strategies,” “build self-confidence,” “increase joy in life,” and “just curious.” Participants were periodically reminded of this goal during the intervention, and it was displayed in the dashboard of the app. During the intervention, participants could also set weekly relaxation goals in terms of a targeted number of relaxation exercises per week (with 1 exercise per week at minimum and a default of 3 exercises per week irrespective of the type of exercise). Weekly coaching sessions with a text-based conversational agent called Lumy provided motivational input for effective and lasting behavior change (integration of relaxation into daily life). Participants were encouraged to set small, realistic relaxation goals for themselves, choose and practice any relaxation exercise at their convenience to meet their goals, and chat with Lumy each week. The minimum expectation for participation in this intervention was completing at least one relaxation exercise and one coaching session per week over the 10-week intervention period.

#### Technical Implementation of the CanRelax App 2.0

**Figure 1:**
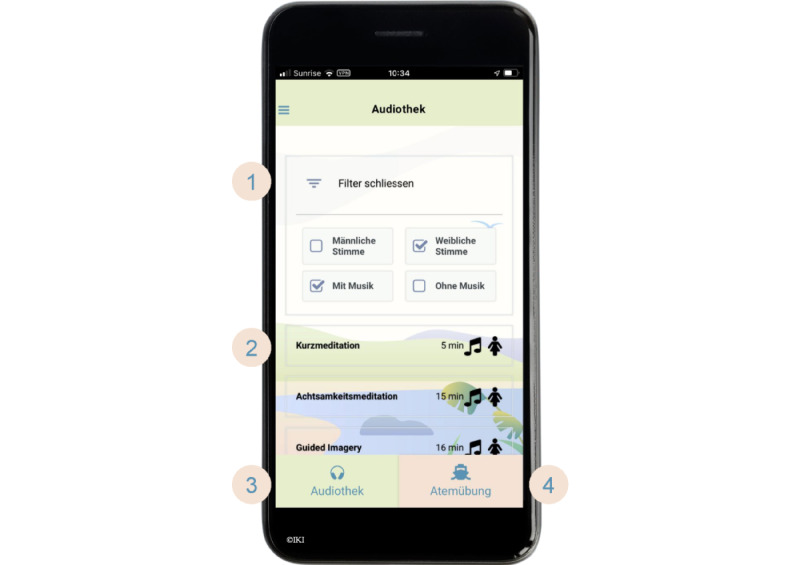
Screenshot of the CanRelax app 2.0—resource library with relaxation exercises. (1) Filter for exercise characteristics (male or female voice with or without background music), (2) search results (can be scrolled for further exercises), (3) audio files, and (4) breathing training.

**Figure 2:**
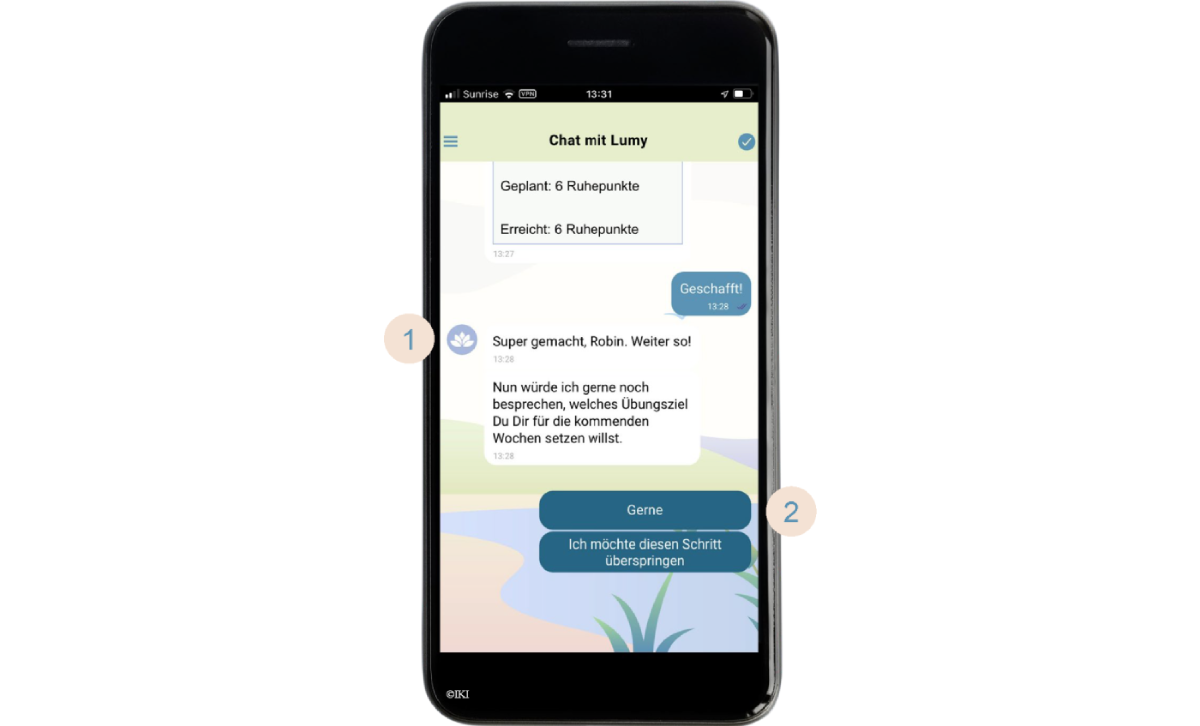
Screenshot of the CanRelax app 2.0—interaction with the conversational agent Lumy (reviewing and adjusting goals). (1) Lumy: “Well done, Robin. Now let’s talk about the goal you want to set for yourself in the coming weeks.” (2) Answer options: “Okay” or “I prefer to skip this part.”.

**Figure 3:**
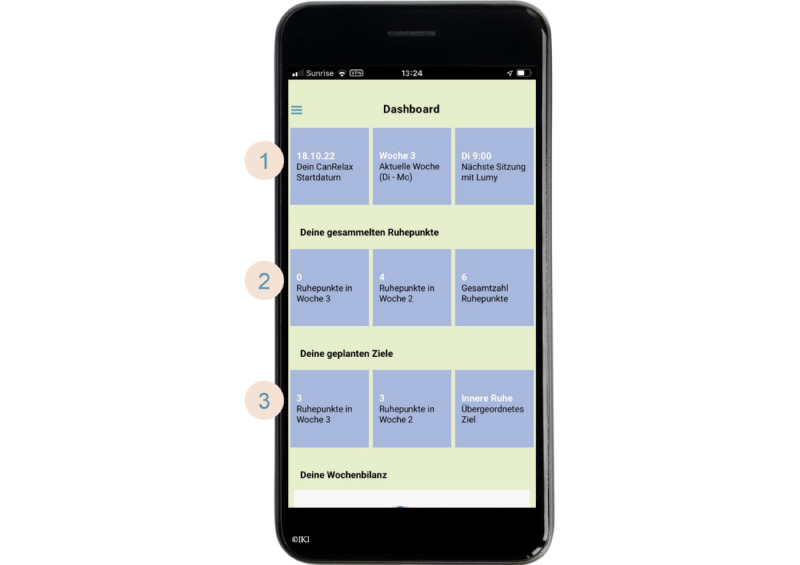
Screenshot of the CanRelax app 2.0—dashboard. (1) Intervention start date, current week, and next chat appointment with Lumy; (2) collected points in the current and previous week and in total; and (3) personal relaxation goals (number of relaxation exercises) in the current and previous week and outcome goal of the participant.

The app was built using MobileCoach (version 21.9.1), an open-source software platform for digital biomarker and health intervention research [32,33]. Conceptually, the app implements the Talk-and-Tools paradigm, which was applied successfully in the domain of mHealth behavior change interventions [34]. The app offers a user interface with a conversational agent (the *talk*) and a broad range of *tools* (Multimedia Appendix 2). Our conversational agent Lumy is visually represented by a neutral (nonhuman) avatar (Multimedia Appendix 3). By choosing a nonhuman avatar, we aimed to create an inclusive experience for all app users and followed best practices and design principles of popular commercial mindfulness and relaxation apps (eg, Headspace). The tools include evidence-based relaxation exercises, a self-monitoring dashboard with metrics on participants’ goals and progress, an educational video clip, personalized in-app letters, frequently asked question (FAQ) sections on the mechanisms and benefits of relaxation as well as on creating healthy habits, and tailored reminder notifications to support regular relaxation practice and engagement with the app. Screenshots of the app can be found in Figures 1-3.

#### Theoretical Principles and Operationalization

##### Overview

**Figure 4:**
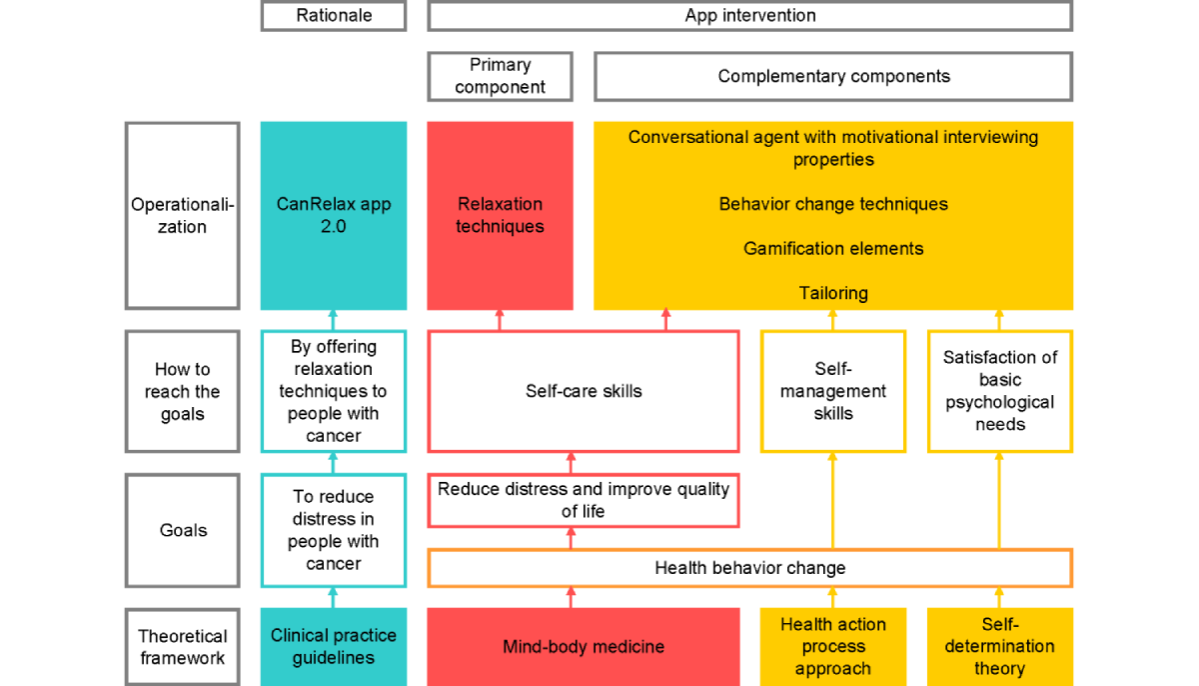
Theoretical framework and operationalization of the CanRelax app 2.0.

The CanRelax app 2.0 implements clinical practice guidelines [35,36]; is grounded in mind-body medicine (MBM) [37,38], the Health Action Process Approach (HAPA) [39], and self-determination theory (SDT) [40]; and includes 39 BCTs (Multimedia Appendix 4 [41]) translated into app features and content. BCTs are active components of behavior change interventions [42] that can influence users’ engagement at both the micro and macro levels. At the micro level, BCTs such as prompts or cues can increase user engagement with the app itself. At the macro level, BCTs can increase engagement with the target behavior (relaxation practice), for example, by using goal setting or self-monitoring features [6,7]. The underlying concept of the intervention flow and the structure of the coaching sessions are informed by generic principles of face-to-face coaching sessions, and we used motivational interviewing (MI) [43,44] aspects as a communication approach. To support the integration of relaxation into everyday routines, we applied the complementing principles of MBM, the HAPA, and SDT as outlined in Figure 4 and detailed in the following sections.

##### Clinical Practice Guidelines

The CanRelax app 2.0 aims to identify and address distress according to clinical practice guideline recommendations on distress management in people with cancer [35,36] by offering a relaxation and mindfulness intervention specifically designed for individuals with cancer, including initial assessment and monitoring of distress using validated tools such as the Distress Thermometer [29].

##### MBM Approach

MBM is a resource-oriented approach centered on empowering individuals and supporting healthy, sustainable behaviors [37,38]. Relaxation and mindfulness are widely used self-care interventions in MBM. The CanRelax app 2.0, being a mind-body intervention, provides the opportunity to learn different relaxation techniques along with educational material on distress during cancer, relaxation, and creating healthy habits.

##### HAPA Framework

Healthy behavior change is at the core of the HAPA. The HAPA focuses on the difficulty of behaving according to one’s intentions and suggests to bridge this intention-behavior gap through perceived self-efficacy, action planning, and coping planning [39]. The CanRelax app 2.0 seeks to enhance self-efficacy and self-management skills through self-enactable BCTs with practical examples of use, such as problem-solving, positive reframing, behavioral experiments, graded tasks, prompts, and self-kindness [41]. Among automatically preselected themes and BCTs (triggered by participants’ interaction with the app), participants can pick the components and topics most relevant to them. The app encourages participants to try new BCTs, determine what works for them, and use these techniques in their daily lives to stay motivated. Participants can also set their own relaxation goals and choose the support they wish to receive from Lumy.

##### SDT Approach

SDT sees healthy behavior change as closely linked to the satisfaction of basic psychological needs for autonomy, competence, and relatedness [40]. The CanRelax app 2.0 supports these basic needs by offering meaningful rationales and choices, using autonomy-supportive language, acknowledging people’s preferences, recognizing their efforts, and promoting a feeling of being cared for through supportive coaching sessions and peer support. Peer support is implemented through personalized letters in the app from semifictional people with cancer sharing their struggles and strategies for overcoming obstacles. Personal preferences are acknowledged, for example, by tailoring emojis to participants’ preferred skin tone and providing all chat content in 3 gender options (woman; man; and a gender-neutral option using the gender star, an asterisk placed within German words such as in “Liebe*r Andrea”). Participants select both the skin tone of their emoji and their preferred gender option during the onboarding process. We also let individuals choose their nickname and a form of address they are comfortable with (formal or informal), showing respect for their personal preferences in relation to language use [45].

##### MI Approach

MI is a person-centered communication approach that relates to the selected behavior change theories in that it aims to create a collaborative environment, draws on people’s own goals and values, and supports their autonomy [43,44]. Examples illustrating the integration of MI principles into the app are provided in the *Coaching Sessions and Tailoring* section.

#### Relaxation Exercises

The app offers 7 different types of relaxation exercises recommended as evidence-based interventions to reduce distress in people with cancer [35,36,46,47]. The relaxation exercises include guided audio recordings of a short meditation (5 minutes), walking meditation (5 minutes), mindfulness meditation (15 minutes), guided imagery (15 minutes), progressive muscle relaxation (15 minutes), body scan (40 minutes), and slow-paced breathing training with visual guidance through gameful visualizations (2-5 minutes; Breeze 2 [48]). The audio files are available in male and female voices with and without background music. The FAQ sidebar submenu in the app provides a selection aid with more information about the different types of relaxation exercises.

#### Self-Monitoring Dashboard With Gamification Elements

The CanRelax app 2.0 tracks relaxation exercises and rewards participants with points as a gamification element. Earned points count toward participants’ self-set weekly relaxation goals. Participants can also earn points by practicing relaxation exercises without the CanRelax app 2.0 (using a different app or without using any app) provided they add this information manually when prompted by Lumy during the coaching sessions. A self-monitoring dashboard illustrates earned points as progress circles. It also provides an overview of the relaxation goals and includes other useful information such as the date and time of the next coaching session.

#### Coaching Sessions and Tailoring

Lumy was developed as a friendly conversational agent that guides participants through the intervention via a series of rule-based, predefined, and personalized conversational turns that simulate the back-and-forth of a real-life conversation. A full coaching session consists of approximately 60 conversational turns (counted in pairs, with one conversational turn consisting of one message from Lumy and one from the participant in response). The conversational flow adapts to the responses chosen by the participants and is enhanced through various ways of tailoring (Textbox 1).

We adopted the structure of a typical face-to-face behavioral coaching session to build the chat sessions in the app [51]. The sessions start with a greeting, followed by small talk about a neutral topic (eg, about the weather) or a “how are you?” sequence and an introduction to the session (including a snooze option to postpone the session). The core part includes assessing the participants’ current state, reviewing previously discussed topics and experiences with BCTs (if applicable), and applying coaching techniques based on MI [43,44]. The implemented techniques focus on building confidence for change (eg, scaling questions, shifting focus away from obstacles and barriers, reframing to offer new and positive interpretations, expressing empathy, affirming, and expressing respect by asking for permission before the conversation starts or before information is shared). After participants have set new relaxation goals, the sessions are summarized to reflect back the main points of the session. An outlook serves as a bridge to the next session, and participants are again encouraged to try out the selected BCTs before the next session (if applicable). The sessions close with the option to adjust the reminder settings and a farewell.

**Implementation of tailoring concepts according to the extended model of tailoring [49].**

**Tailoring concepts and their implementation in the CanRelax app 2.0**

- Feedback: Lumy gives *feedback* on goal setting, goal achievement, and participants’ self-efficacy toward goal achievement. When participants reach their relaxation goals, Lumy celebrates their achievements, and when things do not go well, Lumy tries to offer support.
- Interhuman interaction: in case of urgent need, Lumy encourages *interhuman interaction* through built-in support to contact relevant services that offer advice and support. Inspired by human coaches, we programmed Lumy to show great attention and commitment, listen with curiosity, reflect, and encourage participants to overcome obstacles. When participants report a challenge they came up against in their practice, they have the option to learn about tips and techniques (behavior change techniques [BCTs]) that can help overcome that challenge. They can choose to skip this section or pick a topic they find interesting among 3 preselected BCTs. Selected BCTs are delivered through personalized in-app letters from semifictional peers, which is another way of supporting *interhuman interaction.*
- Adaptation: the BCTs are *adapted* precisely to the reported challenge, and the preselected options are renewed in each coaching session to help keep the sessions interesting.
- User targeting: the concept of *user targeting* attempts to give participants the impression that the conversation was designed especially for them [49]. We incorporated this concept by identifying participants by their nicknames. We also regard participants’ chosen pronouns (formal or informal), gender identity terms, and emojis as expressions of how participants construct their web identity in the context of the CanRelax app 2.0 [50] and match the chat conversations and the app accordingly.
- Goal setting: *goal setting* is a BCT that can be used to tailor an intervention and give participants a feeling of progress over time [49]. In CanRelax 2.0, participants’ own weekly relaxation goals and objectives are at the center of the intervention.
- Context awareness: the tailoring concept *context awareness* aims at providing relevant information considering participants’ (external) situation [49]. We incorporated this by tailoring greeting and farewell messages to the time of day and small talk topics to the season of the year, where applicable.
- Self-learning: CanRelax 2.0 is a *self-learning* app in the sense that it *learns* from the interactions with the participants and updates the intervention accordingly. For example, it records the obstacles that participants report and the BCTs they select and uses this information as a bridge to future sessions. To give continuity, the subsequent coaching sessions take up previously discussed topics and include a recap of experiences and learnings (if any) with the new BCTs between sessions.

#### Iterative Development and Testing

We developed the CanRelax app 2.0 in iterative processes involving input from people with cancer, health professionals, and an interdisciplinary team. The CanRelax app 2.0 builds on a basic app version, which provided relaxation exercises and a reminder function but no other tools or a conversational agent [52]. In version 2.0, we included new features, enhanced functionality, and a solid theory base. During the development process, we conducted usability testing with people with cancer to determine whether they understood and enjoyed the app and whether the app features met their needs. We submitted the usability testing study synopsis to the ethics committee of Zurich, Switzerland, and after review, they stated that the study did not fall under the regulation of the Human Research Act of Switzerland (ethics ID: 2020-00224). A total of 9 individuals with cancer consented to test a prototype of the app, of whom 3 provided detailed feedback, 3 did not test the app in the given time frame, and 3 had technical issues or privacy concerns regarding the test environment. Originally, we planned to conduct the usability tests in person, but due to circumstances related to COVID-19, we had to switch to a fully web-based approach using self-reports. In addition, we thoroughly and repeatedly pretested the app content and features with a multidisciplinary team. The team consisted of professionals with expertise in software engineering, computer science, psychology, psychotherapy, medicine, MBM, nursing, and teaching. Most user feedback was centered on the scripted coaching dialogues with Lumy. We clustered the comments into two main categories and iteratively implemented (1) more variety, in-depth responses, and tailored follow-up questions in the conversation (eg, adjusted the wording of unsatisfactory conversational turns, extended sets of predefined answer options, added links to previously discussed topics, and created unique session openings); and (2) more active choice options with possibilities to skip parts of the conversation, the ability to select topics of personal relevance and interest, the ability to formulate own reminders, and a snooze feature. All improvements were continually refined and tested over 2 years until user satisfaction was achieved.

### Assessments

We collected self-reported data (through Lumy and structured in-app questionnaires) and objective app use data at different time points during the 10-week study period. Only the relevant measures considered for this analysis are described in detail in this paper; the measures of the larger study are reported in Multimedia Appendix 1.

#### Distress and Sociodemographics

At screening and baseline, we collected participants’ self-reported level of distress using a well-known and validated instrument (Distress Thermometer [29]) and sociodemographics such as age, educational attainment, and sex assigned at birth using a structured in-app questionnaire. In the first chat with Lumy, we stored the selected gender identity terms, emoji skin tone modifiers, and preference for formal or informal pronouns (“Du” or “Sie” for “you” in German) to personalize the chat sessions and assessed participants’ initial motivation for downloading the app (outcome goal; 5 forced-choice answer options; see the *Intervention* section).

#### Macrolevel Engagement

To answer the research questions of this paper, we combined engagement data on different levels. Data on macrolevel engagement were gathered in the weekly coaching sessions with Lumy. In each session, we asked about relaxation techniques practiced without using the CanRelax app 2.0. The exact wording changed slightly from week to week to help keep the conversation natural (example wording if at least one relaxation exercise was completed in the app: “Did you practice in any other way last week, besides using the CanRelax app?” If no relaxation exercise was completed in the app, the wording was as follows: “Have you practiced in a different way instead, without the CanRelax app?” An example follow-up question if participants answered “yes” would be the following: “In the past seven days, how often have you practiced without using the CanRelax app?”). We assessed reasons for practicing relaxation exercises without the app (if applicable) once per participant and participants’ self-efficacy toward self-set relaxation goals biweekly using a single-item measure developed with the recommended wording for assessing a specific health behavior [53] (“How confident are you that you will reach your relaxation goal next week, even if it gets difficult?”; participants responded on a visual analog scale implemented as a horizontal slider with values from 0 [*not at all confident*] to 10 [*very confident*]).

#### Microlevel Engagement

At the micro level, we collected participants’ feedback on the app at week 10 with single-choice questions about their favorite feature and the features they would like to change in the app (7 forced-choice answer options in random order) and an option to provide additional information in a free-text field. In addition, the CanRelax app 2.0 tracked the use of different app components (relaxation exercises in the app and coaching sessions with Lumy) over the entire intervention period. Relaxation exercises were considered completed when they were played for 66% of their total run time, and weekly coaching sessions were considered completed when the session closing was reached. We counted the chat sessions 1 to 11 as coaching sessions but not session 0 (onboarding) as completing this session was a requirement for enrollment.

#### Adherence Definition

We used an adherence definition of at least one relaxation exercise or one coaching session per week for 80% of the weeks during the study period to identify participants who complied fully with the app use suggestions.

### Analyses

We conducted descriptive and exploratory data analyses to investigate the data set and thematic analysis of free-text comments. Descriptive statistics were used to report the baseline characteristics of the participants, participants’ self-set goals and self-efficacy, and quantitative in-app feedback. Data visualization methods, supplemented by numerical measures, were used to summarize the main characteristics of the data collected on engagement. We tested for differences in the number of completed relaxation exercises and coaching sessions between prespecified subgroups (distress level at baseline, sex, educational attainment, and age). For this purpose, we conducted a Mann-Whitney *U* test (in the case of 2 groups) or a Kruskal-Wallis test (for >2 groups) after a detailed investigation of descriptive statistics, checking for outliers using box plots and testing normality using a Shapiro-Wilk normality test and *Q*-*Q* plots. Qualitative free-text feedback was analyzed thematically using an inductive approach with the feedback statements as a coding unit, coded into multiple categories where applicable [54].

All analyses were conducted for the entire sample, including those participants who never used the app after onboarding, except for the comparison of relaxation exercises completed using the app versus without using the app. We expected no missing values in baseline variables as completing the questionnaires was a prerequisite for enrollment and participants could not skip questions. Nevertheless, 1 educational attainment response was missing from 1 participant for unknown reasons. Missing values related to the number of exercises or coaching sessions were treated as 0 (no exercise or coaching session completed). Other missing values (educational attainment, self-efficacy, reasons for practicing without the app, and participants’ feedback on the app) were not considered in the analyses.

Statistical analyses and visualizations were conducted using R language (version 4.2.2; R Foundation for Statistical Computing) [55] through RStudio (version 2023.06.0+421; Posit, PBC) [56] using *dplyr* [57] for data manipulation and summary statistics; *ggplot2* [58] for box plots and bar plots; *qqplotr* [59] for *Q*-*Q* plots; *DescTools* [60] for median CIs; and the base R *stats* package to compute the Wilcoxon, Shapiro-Wilks, and Kruskal-Wallis tests.

### Ethical Considerations

We submitted the study synopsis to the ethics committee of Zurich, Switzerland, and after review, they stated that the study did not fall under the regulation of the Human Research Act of Switzerland (ethics ID: 2021-01071). The study was conducted according to the Declaration of Helsinki, the Human Research Act, and the Human Research Ordinance. Informed consent was obtained via the app from each participant before enrollment. All data were collected and stored in secure databases and analyzed in a pseudonymized form. Participants did not receive any compensation. Only participants in the intervention group and the nonrandomized third arm received immediate access to the app’s primary features (ie, the relaxation exercises, weekly coaching sessions with Lumy, BCTs, dashboard, reminder notifications, educational video clip, peer support letters, and FAQs), but everyone who downloaded the app had access to a sidebar submenu with useful links (ie, cancer and mental health information leaflets and links to organizations offering support and counseling) and crisis numbers in case urgent help was needed. By using a rule-based conversational agent, we adopted a highly transparent and safe approach compared to artificial intelligence chatbots and had complete control over the content and flow of the coaching sessions [61-63].

## Results

### Baseline Characteristics

The sample included 77% (77/100) of individuals assigned female at birth and 23% (23/100) of individuals assigned male at birth, and 70% (70/100) self-identified as women, 22% (22/100) self-identified as men, and 8% (8/100) preferred not to disclose their gender. Participants were aged 26 to 79 years (mean 55.6, SD 10.7 years), and 51% (51/100) had a bachelor’s degree or higher. The baseline mean distress level (Distress Thermometer [29]) was 5.6 (SD 2.2), with a mean of 6.9 (SD 1.3) in the high-distress (intervention) group versus 3.1 (SD 0.9) in the low-distress (nonrandomized) group. Baseline characteristics between participants in the high-distress (intervention) group (67/100, 67%) and low-distress (nonrandomized) group (33/100, 33%) were generally comparable except that the high-distress group had fewer participants who installed the app because they were “just curious” (5/67, 7% vs 7/33, 21%). Overall, the most common motivations for installing the app were to improve coping strategies (37/100, 37%) and find inner peace (35/100, 35%; Table 1).

**Table 1:** Descriptive information about the study sample (N=100).

			Total		High-distress group (n=67)		Low-distress group (n=33)
Distress level^a^, mean (SD)			5.6 (2.2)		6.9 (1.3)		3.1 (0.9)
**Sex assigned at birth, n (%)**							
	Female	77 (77)		53 (79)		24 (73)	
	Male	23 (23)		14 (21)		9 (27)	
**Gender, n (%)**							
	Woman	70 (70)		48 (72)		22 (67)	
	Man	22 (22)		13 (19)		9 (27)	
	Other	8 (8)		6 (9)		2 (6)	
**Age (years), n (%)**							
	18-44	12 (12)		8 (12)		4 (12)	
	45-64	68 (68)		47 (70)		21 (64)	
	>64	20 (20)		12 (18)		8 (24)	
**Educational attainment, n (%)**							
	Nontertiary	48 (48)		34 (51)		14 (42)	
	Tertiary	51 (51)		32 (48)		19 (58)	
	Missing	1 (1)		1 (1)		0 (0)	
**Outcome goal, n (%)**							
	Coping resources	37 (37)		26 (39)		11 (33)	
	Inner peace	35 (35)		24 (36)		11 (33)	
	Just curious	12 (12)		5 (7)		7 (21)	
	Joy in life	10 (10)		8 (12)		2 (6)	
	Self-confidence	6 (6)		4 (6)		2 (6)	

^a^Distress measured using the Distress Thermometer [29] with a rating scale ranging from 0 (no distress) to 10 (extreme distress).

### App Engagement

**Figure 5:**
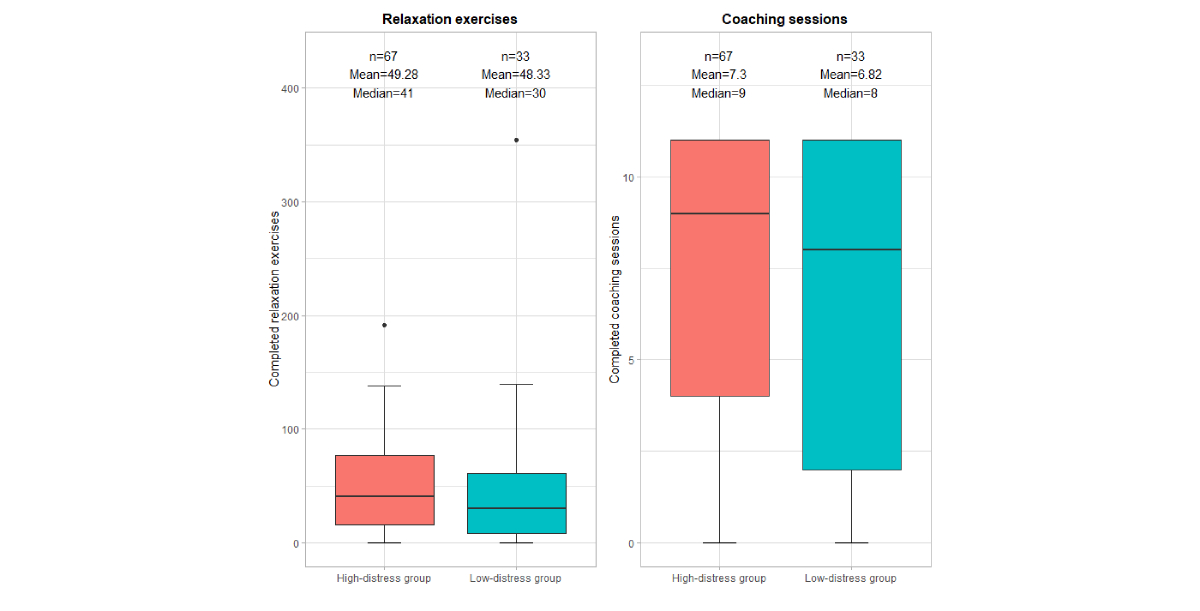
Comparison of completed relaxation exercises and completed coaching sessions in the high-distress group versus the low-distress group (N=100).

**Figure 6:**
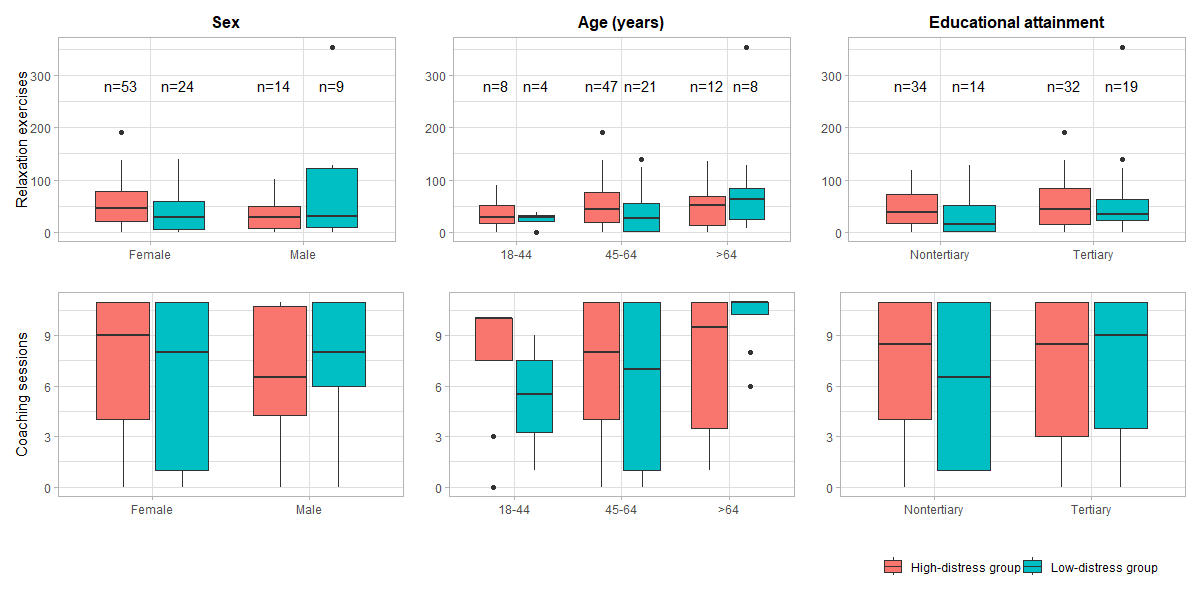
Comparison of completed relaxation exercises and completed coaching sessions in the high-distress group versus the low-distress group for 3 subgroups (N=100).

A visual description of the participants’ app use (completed relaxation exercises and coaching sessions) is presented in Multimedia Appendix 5 and Figures 5 and 6, supplemented by the numerical measures in Table 2. During the 10-week study period, 95% (95/100) of the participants used the app at least once after onboarding. These 95 participants completed a total of 4897 relaxation exercises (median 38, IQR 18-73.5) and 714 coaching sessions (median 9, IQR 4-11) over 10 weeks. Of the total number of relaxation exercises, 71.88% (3520/4897) were completed using the CanRelax app 2.0 (95/100, 95% of the participants; median 25.5, IQR 13-55), and 28.12% (1377/4897) were reported as completed without using the app (median 10, IQR 3-19). Among those participants who reported having completed relaxation exercises without using the app, 28% (21/76) specified that they had used different relaxation recordings, 18% (14/76) did not have their smartphones near them, 16% (12/76) knew the exercises by heart, 4% (3/76) preferred to relax without audio recordings, and 34% (26/76) had other reasons for relaxing without using the CanRelax app 2.0.

The proportion of participants who completed at least one relaxation exercise or one coaching session per week (“active app users”) dropped from 88% (88/100) in the first week to 62% (62/100) in week 10. A total of 64% (64/100) of the participants complied with the app use suggestions per our adherence definition.

Participants’ perceived self-efficacy toward self-set relaxation goals stayed at a median of 8 (0=very low; 10=very high) throughout the 10-week study period, whereas participants raised their relaxation goals. The level of the self-set goals increased from a median of 3 relaxation exercises per week in the first half of the study period (sessions 1 and 3) to a median of 4 exercises per week in the second half (sessions 5, 7, and 9).

App engagement did not vary across prespecified subgroups (ie, distress level at baseline, sex, educational attainment, and age). Mean rank comparisons showed no substantial difference in the number of completed relaxation exercises or coaching sessions among these subgroups (Table 2).

Of the 100 participants, 52 (52%) provided in-app feedback after the 10-week study period (during session 11; Multimedia Appendix 6). A total of 88% (46/52) of the respondents indicated that they “really enjoyed” or “quite enjoyed” chatting with Lumy, and all respondents rated the overall app experience as “very satisfactory” (41/52, 79%) or “quite satisfactory” (11/52, 21%). The favorite app features of the respondents were relaxation exercises (37/52, 71%) and coaching sessions (12/52, 23%). Elements of the app that respondents felt could be improved included “nothing” (29/52, 56%), “something else” than the answer options provided (7/52, 13%), letters from semifictional peers (5/52, 10%), relaxation exercises (4/52, 8%), and in-app questionnaires related to the RCT (4/52, 8%). Of the 52 completed feedback questionnaires, 41 (79%) contained optional free-text comments from participants contextualizing their selected favorite (41 comments) and least favorite (20 comments) app features. Respondents particularly enjoyed the collection of relaxation exercises (12 mentions), liked the format and voices of the exercises (11 mentions), and found that the exercises helped them relax (8 mentions). For example, one respondent stated that the relaxation exercises “are well constructed, with pleasant voices and short.” However, 20% (8/41) of the respondents would have appreciated a wider selection of exercises to choose from. Another main topic that emerged from the analysis was a positive experience of the interaction with Lumy (9 mentions). The coaching sessions were experienced as friendly, uplifting, and encouraging, as seen in the following example:

It is a very friendly chat with a sense of humor, and it always motivates me.

Another respondent appreciated “the conscious reflection and looking back. The feeling of being accompanied and encouraged.” However, 5% (2/41) of the respondents also felt that the interaction with Lumy sounded too robotic or was not interactive enough (1 mention each). Tables S1 and S2 in Multimedia Appendix 7 provide an overview of all free-text comments.

**Table 2:** Use of the CanRelax app 2.0 in the first 100 study participants with immediate access to the app, stratified by subgroup (N=100).

				Participants, n (%)		Completed relaxation exercises over 10 weeks				Completed coaching sessions over 10 weeks		
						Values, median (IQR)		*P* value^a^		Values, median (IQR)		*P* value^a^
Entire sample				100 (100)		34.5 (14-70.75)		—^b^		8 (4-11)		—
**Subgroup**												
	**Distress**											
		High	67 (67)		41 (15.5-77)		.21		9 (4-11)		.84	
		Low	33 (33)		30 (8-61)		—		8 (2-11)		—	
	**Sex assigned at birth**											
		Female	77 (77)		40 (15-70)		.47		9 (3-11)		.64	
		Male	23 (23)		30 (8.5-64.5)		—		7 (4.5-11)		—	
	**Educational attainment^c^**											
		Nontertiary	48 (48)		31.5 (12.75-64.75)		.20		8 (2.75-11)		.49	
		Tertiary	51 (51)		41 (18-78)		—		9 (3-11)		—	
	**Age group (years)**											
		18-44	11 (11)		27 (12.5-39.5)		.34		9 (3.5-10)		.16	
		45-64	68 (68)		34.5 (13.25-73.25)		—		8 (3-11)		—	
		>64	20 (20)		52.5 (19.25-72.25)		—		11 (6.75-11)		—	

^a^2-sided *P* values derived from the Mann-Whitney *U* test (distress, sex, and educational attainment) and Kruskal-Wallis test (age group).

^b^Not applicable.

^c^1 missing value.

## Discussion

### Principal Findings

Overall, engagement with the CanRelax app 2.0 declined over the study period but stayed relatively high, with 62% (62/100) of participants actively using the app in week 10. Engagement was unrelated to participant characteristics such as level of distress at baseline, sex assigned at birth, educational attainment, or age. More than one-quarter (1377/4897, 28.12%) of the relaxation exercises were completed without using the app, supporting the need for assessing engagement on a macro level. Participants’ self-efficacy remained stable at a high level. At the same time, participants raised their relaxation goals, which indicates a potential relative increase in self-efficacy. Participants who completed the intervention highly valued the app. Free-text comments suggested that a wider variety of relaxation exercises would further enhance the user experience.

### Comparison With Prior Work

Engagement rates with cancer-related digital interventions tend to be higher than in other populations, but high variability in engagement measures and intervention components and lack of a threshold for acceptable engagement make it difficult to compare findings across studies. Reviews of empirical studies using cancer-related digital interventions have reported use rates between 70% and 100% [64,65]. These high use rates contrast with the generally low engagement with mHealth apps reported for individuals with other health conditions [2,10,11,13-15] and suggest that people with cancer might be particularly inclined to improve their health and change certain health behaviors through mHealth apps. Stressful life events such as the diagnosis and treatment of cancer potentially serve as catalysts for behavior change [66,67]. Nonetheless, comparing engagement across studies is difficult as there are no standards regarding the assessment, reporting, and interpretation of engagement with mHealth apps. In a recent review, every primary study stated that their apps achieved good engagement despite large differences in criteria used to assess engagement and a range of reported engagement rates from 35% to 100% [16]. This shows an urgent need for standards for assessing, reporting, and interpreting engagement with mHealth apps [16].

Fully automated mHealth studies with no human support are prone to low engagement rates, but there is great potential for increasing engagement using fully automated social components, behavior change theory, and design principles of successful commercial apps. Most mHealth apps in research settings provide human support, whereas popular commercial apps are typically unguided. Human support is known to positively influence engagement and effectiveness but drastically limits the scalability of mHealth apps [3,18]. Despite this limitation, most mHealth apps in research settings provide human support at varying levels—from high support through guided interventions (ie, involving guidance from a trained professional, eg, through live videoconferencing or web-based workshops) to lower levels of support through study processes (eg, screening visits or telephone surveys conducted by the study team). In the rare studies available on unguided cancer-related mHealth apps with no human support [68-70], engagement rates were <50%. One reason could be that existing researcher-developed apps are not engaging enough and, therefore, need human support to motivate participants [18,71]. This may be less the case for popular commercial apps, which are typically unguided (eg, Headspace and Calm) [18]. Thus, there is great potential for unguided research apps to improve user engagement and the generalizability of research findings to real-life settings if they learn from successful commercial apps. An example of an mHealth study with a low level of human support is the CanRelax 1.0 feasibility study [52]. The CanRelax app 1.0 was a fully automated mHealth app, but study processes such as enrollment were supported by study staff. The authors classified 54% (54/100) of participants as continuous app users in week 10 [52]. In comparison, engagement with the enhanced CanRelax app 2.0 in week 10 improved to 62% (62/100) even though we provided no human support and used stricter definitions of engagement. It is possible that the fully automated social components in the CanRelax app 2.0, such as the weekly coaching sessions with Lumy, compensated for the lack of human support. This aligns with recent research underscoring the potential of conversational agents to positively impact engagement with mHealth apps [3,72-77]. We demonstrated this potential by combining a conversational agent with a theoretical foundation and incorporating key design principles inspired by highly engaging commercial apps (eg, inclusive avatar and visuals).

Existing findings on the impact of participant characteristics on engagement are inconsistent [78]. In our analyses, engagement was not associated with the demographics (sex assigned at birth, educational attainment, and age) or psychological characteristics (level of distress) of the participants. These results contradict the findings of earlier studies that showed higher engagement in female individuals [6,52], individuals with higher educational attainment [6,15], younger [15] or older individuals [6], and individuals with higher baseline distress [15,52]. In the CanRelax app 2.0, the content and design features implemented to increase engagement might have succeeded in reaching those groups of people who needed a little extra encouragement and possibly helped level out differences in engagement among subgroups. Given the inconsistencies in the literature, identifying participant characteristics and other factors that influence engagement is an exciting topic for future studies.

Our findings support the feasibility and value of assessing macrolevel engagement in mHealth behavior change interventions. Although the conceptualization of engagement as a multifaceted construct is widely accepted, macrolevel engagement is rarely assessed in mHealth app studies. We approached this gap by examining engagement on multiple levels and showed considerable engagement with the target behavior (ie, relaxation) beyond app use. First, nearly one-third of all completed relaxation exercises (1377/4897, 28.12%) were practiced without using the CanRelax app 2.0. Relaxation techniques can be practiced in different ways depending on one’s experiences, needs, and preferences; for example, beginners could start with guided relaxation via audio recordings (or in-person sessions) and later move on to more silent, self-guided relaxation exercises. In our study, examining only those exercises practiced using the app would have given an incomplete and potentially misleading picture of participants’ engagement with relaxation practices. Second, median self-efficacy remained high even as relaxation goals increased, indicating that participants felt encouraged to tackle challenging tasks and were engaged in the behavior change process [24].

Data on macrolevel engagement are necessary to understand how engagement with an mHealth app changes over time and how these engagement patterns relate to the intended health outcomes. Baglione et al [17] found that high baseline distress was associated with initially higher engagement that declined over time, whereas the engagement of the group of participants with lower baseline distress increased over the course of a 7-week intervention, resulting in similar engagement levels in both groups at week 7. Siebenhüner et al [79] examined the associations between distress and adherence (ie, app use) in the CanRelax app 1.0 and showed that a decrease in the level of distress over time (ie, an improvement in health outcomes) was associated with lower adherence. However, the authors did not assess engagement with the target behavior in daily life. Without this information, it remains unclear whether participants with improved distress stopped using the app because they disengaged from the intervention or no longer needed the app’s support to continue the new behavior [5]. As lower app use could be associated with higher engagement at the macro level, the suggested “adherence benefit paradox” [79] might not be a paradox after all but could even be considered the goal of a successful mHealth app [2].

### Limitations

Our study is subject to common sources of bias that can affect the internal validity and generalizability of the findings. One potential source of bias is the use of self-reported data. To mitigate potential self-reporting bias, we combined self-reported and objectively tracked data in the assessment of engagement. Feedback was only collected from participants who completed the coaching session with Lumy in week 11. As it is possible that only those who enjoyed the app completed this session, feedback might be positively biased. Another potential source of bias is selection bias as our study focused on a group of highly motivated participants. Initial motivation for study participation was needed as participants had no contact with the research team but self-downloaded the app and self-enrolled in the study if they fulfilled the inclusion criteria. Selection bias is also indicated by female individuals being overrepresented in our sample. To improve the generalizability of our study, we used broad recruitment strategies and successfully recruited participants with lower than tertiary education. We also abstained from using research strategies to increase motivation and engagement (eg, compensation for study participation) that would differ from usual real-world app use settings. Another limitation is that we did not consider past engagement with relaxation in our analyses. Participants could have already established a regular relaxation practice before the study; still, engagement with a new app is not necessarily linked to previous experience with relaxation. A third limitation is due to technical issues with the CanRelax app 2.0 during the study, which could have reduced engagement. For example, we did not provide an easy solution to transfer the CanRelax app 2.0 to a new smartphone. Participants with new smartphones had to reach out for technical support and usually had to wait several weeks until they could continue to use the app where they left off. To avoid this problem, individuals must create an account in the future.

### Clinical Implications

For a positive impact on health outcomes on a large scale, mHealth apps need to be scalable, engaging to users, and effective. Scalability is a great advantage of fully automated mHealth apps, but these apps tend to suffer from low engagement rates threatening their effectiveness. Our findings show that successful engagement can be achieved with fully automated mHealth apps that are highly tailored, include fully automated social components and BCTs based on theory and evidence, and are developed with design principles used by popular commercial apps. These results provide a valuable context for subsequent outcome evaluations and add to research on optimizing fully automated digital health interventions.

### Conclusions

The CanRelax app 2.0 achieved similar engagement to that of other cancer-related mHealth apps even though we used stricter criteria for engagement than other studies and provided no human support. The implemented theory- and evidence-based design principles and fully automated social components, such as a conversational agent that simulated human support, might have compensated for the lack of human involvement and contributed to enhanced engagement at both a micro and a macro level. Our findings underline that engagement is a complex and multifaceted construct and that measures at the macro level are particularly valuable to assess engagement not only with the app itself but also with the larger target behavior, which is, ultimately, the goal of an mHealth app.

## Appendix

Multimedia Appendix 1 CanRelax randomized controlled trial study design and assessments.

Multimedia Appendix 2 CanRelax coaching structure.

Multimedia Appendix 3 CanRelax avatar icon.

Multimedia Appendix 4 Behavior change techniques in the CanRelax app 2.0.

Multimedia Appendix 5 Active app users over 10 weeks (N=100).

Multimedia Appendix 6 CanRelax in-app feedback.

Multimedia Appendix 7 CanRelax free-text feedback.

## Abbreviations

BCT: behavior change technique
FAQ: frequently asked question
HAPA: Health Action Process Approach
MBM: mind-body medicine
mHealth: mobile health
MI: motivational interviewing
RCT: randomized controlled trial
SDT: self-determination theory
