# Supplementary material for: Engagement With a Relaxation and Mindfulness Mobile App Among People With Cancer: Exploratory Analysis of Use Data and Self-Reports From a Randomized Controlled Trial
Source: JMIR Cancer. 2024 May 31;10:e52386. doi: 10.2196/52386 (PMC11179041; doi:10.2196/52386)
Supplement: Multimedia Appendix 1 [file cancer_v10i1e52386_app1.pdf]

CanRelax RCT study design and assessments.

Figure S1

CanRelax study design and assessments.

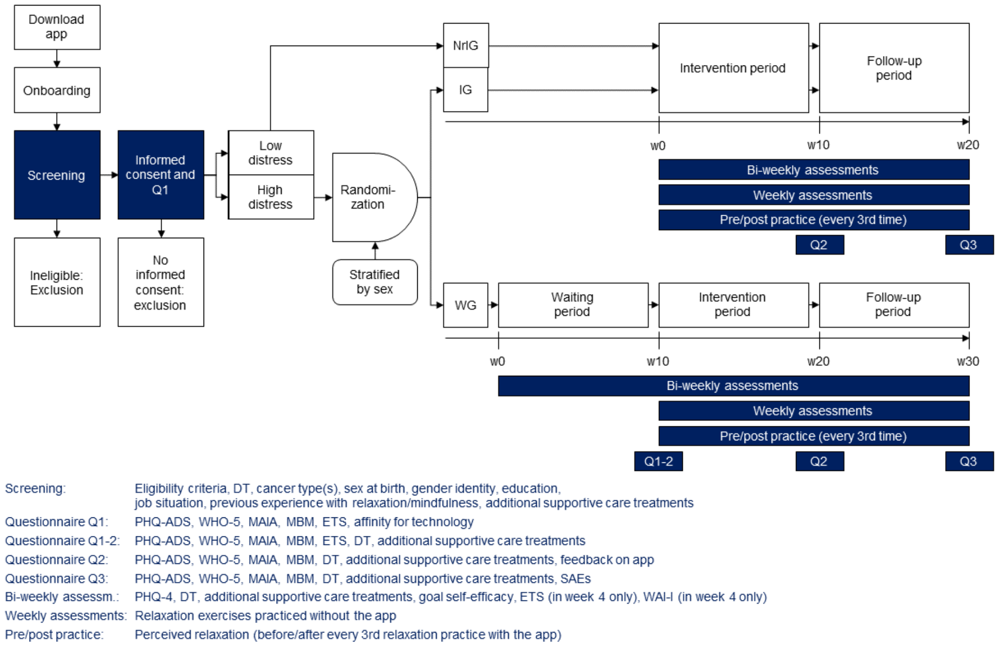

DT: Distress Thermometer [1]; ETS: Expectation for Treatment Scale [2]; IG: Intervention group; NrlG: Nonrandomized intervention group; MAIA: Multidimensional Assessment of Interoceptive Awareness [3] (subscale on self-regulation); MBM: Mind-body medicine practice scale (developed by our institute; selected items on relaxation, mindfulness, and self-care); PHQ-ADS: Patient health questionnaire anxiety and depression scale [4]; PHQ-4: Patient Health Questionnaire-4 [5]; SAEs: Serious adverse events (structured questionnaire); WAI-I: Working Alliance Inventory for guided Internet interventions [6] (adapted for our intervention); WG: Waiting-list control group; WHO-5: Well-being Index [7].

References

1. Mehnert A, Müller D, Lehmann C, Koch U. Die deutsche Version des NCCN Distress-Thermometers. Zeitschrift für Psychiatrie, Psychologie und Psychotherapie. 2006 2006/01/01;54(3):213-223. doi: 10.1024/1661-4747.54.3.213.
2. Barth J, Kern A, Lüthi S, Witt CM. Assessment of patients' expectations: development and validation of the Expectation for Treatment Scale (ETS). BMJ Open. 2019;9(6):e026712. PMID: 31213446. doi: 10.1136/bmjopen-2018-026712.
3. Mehling WE, Price C, Daubenmier JJ, Acree M, Bartmess E, Stewart A. The Multidimensional Assessment of Interoceptive Awareness (MAIA). PLOS ONE. 2012;7(11):e48230. PMID: 23133619. doi: 10.1371/journal.pone.0048230.
4. Kroenke K, Baye F, Lourens SG. Comparative validity and responsiveness of PHQ-ADS and other composite anxiety-depression measures. J Affect Disord. 2019 Mar 1;246:437-443. PMID: 30599366. doi: 10.1016/j.jad.2018.12.098.
5. Kroenke K, Spitzer RL, Williams JBW, Löwe B. An Ultra-Brief Screening Scale for Anxiety and Depression: The PHQ–4. Psychosomatics. 2009 2009/11/01/;50(6):613-621. PMID: 19996233. doi: 10.1176/appi.psy.50.6.613.
6. Gómez Penedo JM, Berger T, Grosse Holtforth M, Krieger T, Schröder J, Hohagen F, et al. The Working Alliance Inventory for guided Internet interventions (WAI‐I). Journal of Clinical Psychology. 2020;76(6):973-986. PMID: 31240727. doi: 10.1002/jclp.22823.
7. Bech P, Olsen LR, Kjoller M, Rasmussen NK. Measuring well-being rather than the absence of distress symptoms: a comparison of the SF-36 Mental Health subscale and the WHO-Five well-being scale. International Journal of Methods in Psychiatric Research. 2003 2003/06/01;12(2):85-91. doi: https://doi.org/10.1002/mpr.145.
