# Supplementary figures and images for: Engagement With a Relaxation and Mindfulness Mobile App Among People With Cancer: Exploratory Analysis of Use Data and Self-Reports From a Randomized Controlled Trial
Source: JMIR Cancer. 2024 May 31;10:e52386. doi: 10.2196/52386 (PMC11179041; doi:10.2196/52386)

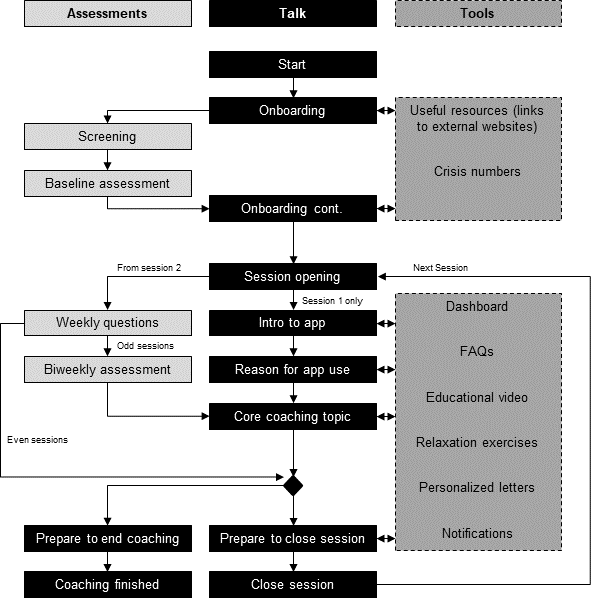

Supplement: Multimedia Appendix 2 [file cancer_v10i1e52386_app2.png]

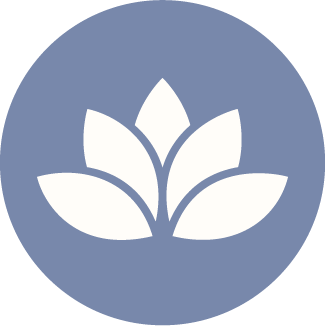

Supplement: Multimedia Appendix 3 [file cancer_v10i1e52386_app3.png]

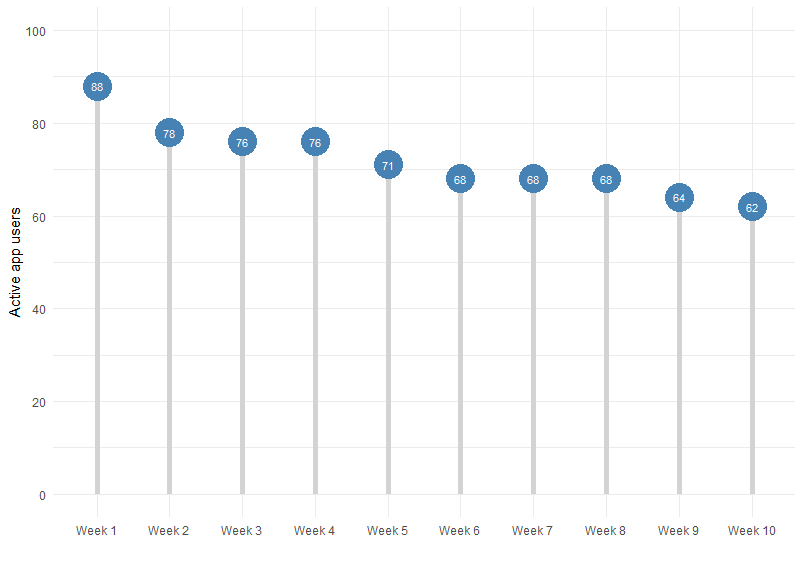

Supplement: Multimedia Appendix 5 [file cancer_v10i1e52386_app5.png]

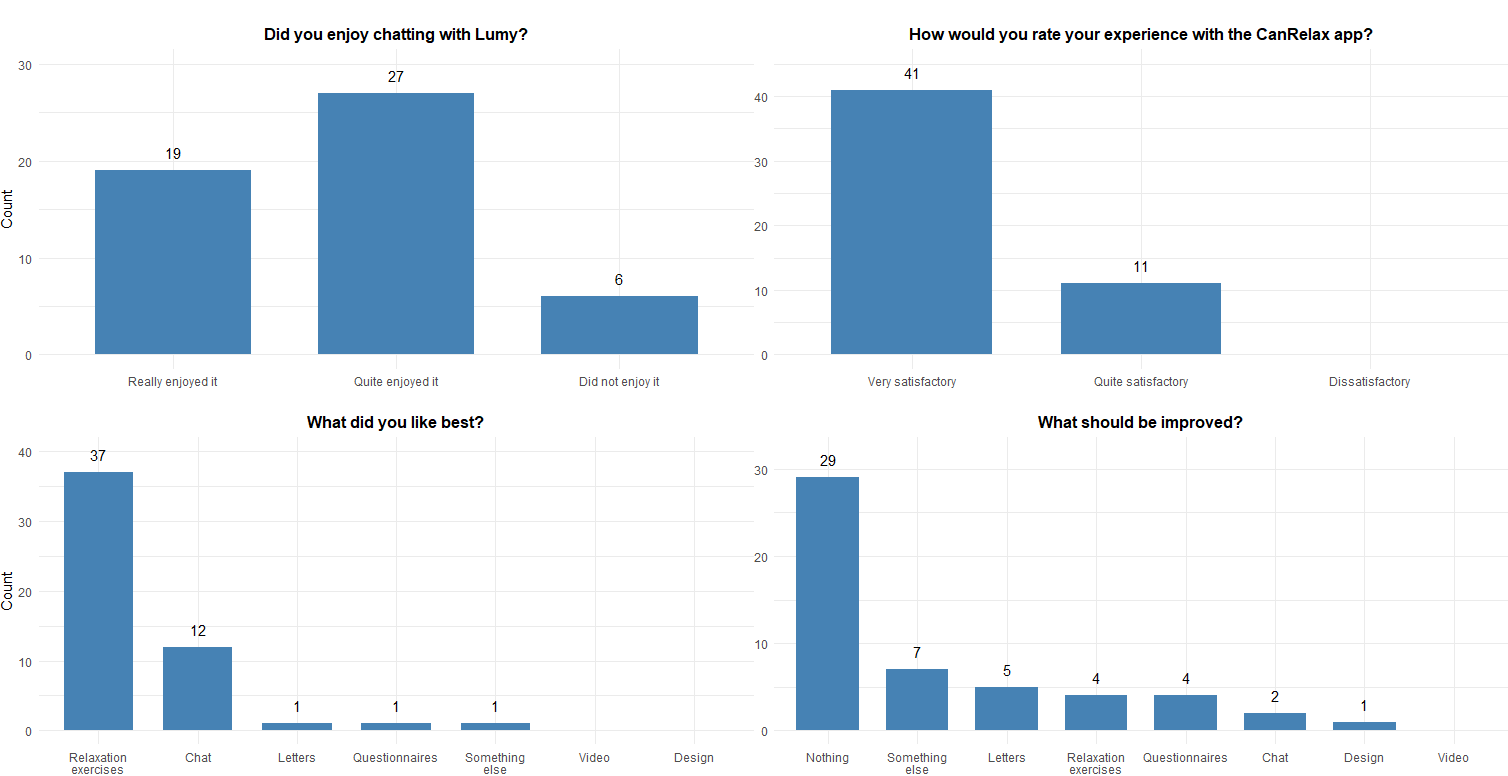

Supplement: Multimedia Appendix 6 [file cancer_v10i1e52386_app6.png]
