# Supplementary material for: Engagement With a Relaxation and Mindfulness Mobile App Among People With Cancer: Exploratory Analysis of Use Data and Self-Reports From a Randomized Controlled Trial
Source: JMIR Cancer. 2024 May 31;10:e52386. doi: 10.2196/52386 (PMC11179041; doi:10.2196/52386)
Supplement: Multimedia Appendix 4 [file cancer_v10i1e52386_app4.pdf]

Behavior change techniques in the CanRelax app 2.0.

Table S1

Behavior change techniques integrated in the CanRelax app 2.0 with technique number, label and definition as per Knittle et al [1]

No.	Label	Definition	App integration
5	Behavioral goal setting	Set a goal defined in terms of the target behavior	Coaching sessions
6	Outcome goal setting	Set a goal defined in terms of a positive outcome of the target behavior	Coaching sessions
7	Problem Solving	Analyze factors influencing the behavior and generate or select strategies to overcome barriers to performing the behavior	Coaching sessions
8	Action planning	Plan performance of behavior in detail, including context, frequency, duration and/or intensity	Coaching sessions
9	Review behavioral goal(s)	Review behavioral goal(s) and consider modifying in light of achievement	Coaching sessions
11	Discrepancy between current behavior and goal	Identify discrepancies between your current behavior and your previously set outcome goals, behavioral goals or action plans	Coaching sessions & Dashboard
12	Self-commitment	Pledge to or promise yourself to perform the target behavior	Personalized in-app letters from semifictional peers
13	Public commitment	Make a commitment to performing the target behavior and inform others about it	Personalized in-app letters from semifictional peers
14	Make a behavioral contract	Create a written specification of the behavior to be performed, and have it witnessed by another person	Personalized in-app letters from semifictional peers
17	Self-monitoring of behavior	Monitor and record your own behavior	Dashboard

19	Monitoring of emotional consequences	Assess feelings after attempts at performing the target behavior	Perceived relaxation ratings before and after the exercises
24	Adding objects to the environment	Add or acquire equipment or objects that facilitate performance of the target behavior	Personalized in-app letters from semifictional peers
25	Restructuring the physical environment	Change the physical environment in order to facilitate performance of the target behavior or create barriers to the unwanted behavior	Personalized in-app letters from semifictional peers
29	Task crafting (enjoyment)	Restructure the target behavior to make performing it more enjoyable	Personalized in-app letters from semifictional peers; Breeze breathing training
32	Goal integration	Modify (or choose ways of doing) the behavior such that it allows for simultaneously engaging in other valued behaviors and/or pursuing valued outcomes	Personalized in-app letters from semifictional peers
33	Behavioral experiments	Identify and test hypotheses about the behavior, its causes and consequences by collecting and interpreting data about such experiences	Personalized in-app letters from semifictional peers
39	Credible source	Watch or listen to a credible source argue in favor of or against the target behavior	Educational video
41	Memory aids	Use techniques to improve recall of information related to carrying out the target behavior	Guided audio-recordings
54	Obtain practical social support	Obtain practical help for performance of the behavior	Personalized in-app letters from semifictional peers
55	Obtain emotional social support	Obtain emotional social support for performance of the behavior	Personalized in-app letters from semifictional peers
56	Obtain instruction on how to perform the behavior	Obtain instruction to learn how to perform the behavior	Guided audio-recordings

57	Remind of outcome goal content	Remind yourself of your outcome goal(s)	Personalized in-app letters from semifictional peers & Dashboard
58	Action control (keep goals in mind)	Make efforts to consciously keep the target behavior and your goals in mind	Personalized in-app letters from semifictional peers & Dashboard
60	Prompts/cues	Introduce environmental or social prompts, cues and reminders to undertake the target behavior	Personalized in-app letters from semifictional peers & Notification Feature
67	Behavior substitution	Substitute an unwanted behavior with a wanted or neutral behavior	Personalized in-app letters from semifictional peers
70	Habit formation	Rehearse and repeat the behavior in the same context repeatedly so that the context automatically elicits the behavior	Personalized in-app letters from semifictional peers
74	Graded tasks	Set easy-to-perform, graded tasks making them increasingly difficult, but achievable, until target behavior is performed	Personalized in-app letters from semifictional peers; Breeze breathing training and guided audio-recordings
80	Self-reward (behavior)	Reward yourself with money, vouchers, experiences or other valued objects if and only if you have made effort or progress in performing the behavior	Personalized in-app letters from semifictional peers
81	Self-praise (behavior)	Reward yourself with a verbal reward if and only if you have made effort or progress in performing the behavior	Personalized in-app letters from semifictional peers
102	Find meaning in target behavior	Identify (or remind yourself of) links between the target behavior and your values, relating the behavior to something personally or socially meaningful	Personalized in-app letters from semifictional peers
105	Associate identity with changed behavior	Construct a new self-identity that is in line with performing the target behavior	Personalized in-app letters from semifictional peers

107	Verbal self-persuasion about own capability	Persuade self of capability to perform target behavior, arguing against self-doubt	Personalized in-app letters from semifictional peers
109	Focus on enjoyment (pleasant aspects) of behavior	Focus thinking on pleasant rather than unpleasant aspects of the target behavior	Personalized in-app letters from semifictional peers
110	Positive reframing	Take a more positive view of your current situation	Personalized in-app letters from semifictional peers & Coaching sessions
113	Manage negative emotions	Take steps to reduce negative emotions to facilitate performance of the behavior	Personalized in-app letters from semifictional peers
114	Normalize difficulty	Recognize (or remind yourself) that it is common to face difficulties when pursuing behavioral changes	Personalized in-app letters from semifictional peers & Coaching sessions
115	Self-kindness	Reflect on your own difficulties, efforts and self-worth, in a kind and accepting way	Personalized in-app letters from semifictional peers
116	Acceptance	Recognize and accept negative thoughts, feelings, emotions, and physiological states related to the target behavior	Personalized in-app letters from semifictional peers
122	Emphasize autonomy	Acknowledge own ability for choice in relation to the target behavior	Personalized in-app letters from semifictional peers & Coaching sessions

References

1. Knittle K, Heino M, Marques MM, Stenius M, Beattie M, Ehbrecht F, et al. The compendium of self-enactable techniques to change and self-manage motivation and behaviour v.1.0. Nat Hum Behav. 2020 Feb;4(2):215-223. PMID: 31932687. doi: 10.1038/s41562-019-0798-9.
