# Supplementary material for: Engagement With a Relaxation and Mindfulness Mobile App Among People With Cancer: Exploratory Analysis of Use Data and Self-Reports From a Randomized Controlled Trial
Source: JMIR Cancer. 2024 May 31;10:e52386. doi: 10.2196/52386 (PMC11179041; doi:10.2196/52386)
Supplement: Multimedia Appendix 7 [file cancer_v10i1e52386_app7.pdf]

## CanRelax app 2.0 free-text feedback.

**Table S1**

*Favorite App Features (Single Choice) and Associated Free-Text Comments Sorted by Themes (Number of Mentions)*

| What did you like best? | |
|---|---|
| **Relaxation exercises** | **Collection, variety (12)**<br>– *Different lengths and the variety*<br>– *The choice. Carefully compiled exercises. Voices are pleasant.* [a]<br>– *That there are different exercises of varying lengths*<br>– *The different ways to relax from 5 min to 40 min. The differentiation with voice and music.* [a]<br>– *There are different exercises I can choose from and different voices.*<br>– *The different lengths of the exercises, depending on the available time one can do a longer/shorter exercise*<br>– *The whole collection is good* [a]<br>– *The choice of different relaxation exercises*<br>– *Everything. That I can choose between several options.*<br>– *Variety, beautiful exercises* [a]<br>– *There are short and longer exercises and one can choose*<br>– *Choice of different exercises*<br>**Exercise characteristics (11)**<br>– *They are rather short, clearly explained exercises, which can be easily integrated into everyday life.* [a]<br>– *Guidance/relaxation support* [a]<br>– *I like the voice that guides me. And I feel relaxed after the exercises.* [a]<br>– *They are well constructed, with pleasant voices and short.*<br>– *Pleasant voice and music.*<br>– *The choice. Carefully compiled exercises. Voices are pleasant.* [a]<br>– *The different ways to relax from 5 min to 40 min. The differentiation with voice and music.* [a] |

| | |
|---|---|
| | – *The whole collection is good* [a]<br>– *Pleasant accompanying voice, clearly structured exercise instructions*<br>– *The structure*<br>– *Variety, beautiful exercises* [a]<br>**Relaxation support (8)**<br>– *Guidance/relaxation support* [a]<br>– *I like the voice that guides me. And I feel relaxed after the exercises* [a]<br>– *The success that these exercises bring.*<br>– *The conscious moments of rest*<br>– *I can access them anytime I need them, they really help me!* [a]<br>– *I do them as a cool down after my gymnastics, but also before falling asleep. I find them very calming.*<br>– *They are effective, I often fall asleep when I do them*<br>– *I find calm, though not for long.*<br>**Building a routine, integration into daily life (4)**<br>– *They are rather short, clearly explained exercises, which can be easily integrated into everyday life.* [a]<br>– *It gives a routine*<br>– *The incentive to relax regularly.*<br>– *Consciously making time for it*<br>**Availability (1)**<br>– *I can access them anytime I need them, they really help me!* [a]<br>**Other (1)**<br>– *I find short exercises especially useful. For example, from eurythmy therapy, I know an exercise for anxiety and stress. Close your fingers to a loose fist when inhaling, open your hand when exhaling, can also be combined with a lip brake. Something short like this can be used everywhere, even before a doctor's visit.* |
| **Chat (ie, coaching sessions with Lumy)** | **Session characteristics (9)**<br>– *The conscious reflection and looking back. The feeling of being accompanied and encouraged*<br>– *They are funny, empathetically future-focused yet specific*<br>– *The back and forth* |

| | |
|---|---|
| | – *Personal, short, comprehensible*<br>– *The interest*<br>– *It is a very friendly chat with a sense of humor, and it always motivates me*<br>– *The questions cheer me up and have the effect that I free up some space for myself despite my busy everyday life.*<br>– *Someone thinks about me and takes me seriously*<br>– *The positive chats* |
| **Questionnaires** | – *They help with self-reflection* |
| **Letters** | – *Helpful tips for daily life* |
| **Something else** | – *Text notifications* |

[a] associated with more than one theme

**Table S2**

*Least Favorite App Features (Single Choice) and Associated Free-Text Comments Sorted by Themes (Number of Mentions)*

| **What should be improved?** | |
|---|---|
| **Relaxation exercises** | **Collection, variety (8)**<br>– *The exercises are all good. A larger selection would be helpful with different lengths*<br>– *Larger selection*<br>– *Variations of the 5 and 15 min. breathing exercises simply for the sake of variety (it's just a machine) empathy is missing, sure* [a]<br>– *A) Everything is ALWAYS positive. B) You can feel the robot behind it. And about the exercises: a little more diversity would be nice.* [a/b]<br>– *Even more exercises would be cool*[c]<br>– *I would like to have a larger range of exercises. Maybe an introduction to meditation?* [c]<br>– *I would like to have more variety in the relaxation exercises. A larger selection, not only with or without music, respectively male or female voice*[c]<br>– *Variety in the relaxation exercises*[c] |

| | |
|---|---|
| | **Exercise characteristics (4)**<br>– *Variations of the 5 and 15 min. breathing exercises simply for the sake of variety (it's just a machine) empathy is missing, sure* [a]<br>– *The tone and the content. Anna Heinrich* [e] *is much better!*<br>– *The breathing exercises app*<br>– *In the Bodyscan I find the inhale and exhale through the crown chakra strange, I have distanced myself from such things and "landed" through my illness with the Father, Son and Holy Spirit. This gives me support and confidence. The design of the app is not relevant for me. I have not seen a video, or at least I can't remember.* |
| **Chat (ie, coaching sessions with Lumy)** | **Session characteristics (2)**<br>– *little interactive*<br>– *A) Everything is ALWAYS positive. B) You can feel the robot behind it. And about the exercises: a little more diversity would be nice.* [a]<br>**Not specified (1)**<br>– *And the chats* [a/d] |
| **Letters** | **Artificial, predictable (3)**<br>– *What is written is a little predictable*<br>– *I find the fictitious senders a bit far-fetched, but that is of course my subjective taste.*<br>– *The letters seem a bit artificial, constructed*<br>**Other (1)**<br>– *I have no entries yet* |
| **Questionnaires** | **Not individualized (2)**<br>– *No space for individual comments*<br>– *They do not take into account external circumstances (eg, when you are on vacation, you are automatically more relaxed).*<br>**Not specified (1)**<br>– *And the chats* [a] |
| **General comment** | – *It would be nice to write something actively from time to time. Eg, when you're on vacation and do not have time to answer* [c] |

[a] associated with more than one theme

[b] least favorite app feature: “chat”
[c] least favorite app feature: “something else”
[d] least favorite app feature: “questionnaires”
[e] Pseudonym
